# Is Smoking a Sin?—Mr. Spurgeon’s Opinion

**Published:** 1874-12

**Authors:** 


Is Smoking a Sin?—The eminent Rev. Mr. Spurgeon has discussed lately another view of the subject. He says:

I demur altogether and most positively to the statement that to smoke tobacco is in itself a sin. It may become so, as any other indifferent action may; but as an action it is no sin. Together with hundreds of thousands of my fellow-Christians I have smoked, and with them I am under the condemnation of living in habitual sin, if certain accusers are to be believed. As I would not knowingly live even in the smallest violation of the law of God, and sin in the transgression of the law, I will not own to sin when I am not conscious of it. There is growing up in society a Pharisaic system which adds to the commands of God the precepts of men; to that system I will not yield for an hour. The preservation of my liberty may bring upon me the upbraiding of many of the good, and the sneers of the self-righteous; but I shall endure both with serenity, so long as I feel clear in my conscience before God.

The expression, "smoking to the glory of God," standing alone, has an ill sound, and I do not justify it; but in the sense in which I employed it, I still stand to it. No Christian should do anything in which he cannot glorify God; and this may be done, according to Scripture, in eating and drinking, and the common actions of life. When I have found intense pain relieved, a weary brain soothed, and calm, refreshing sleep obtained by a cigar, I have felt grateful to God, and have blessed His name. This is what I meant, and by no means did I use sacred words triflingly. If through smoking I had wasted an hour of my time, if I had stinted my gifts to the poor, if I had rendered my mind less vigorous, I trust I should see my fault and turn from it; but he who charges me with these things shall have no answer but my forgiveness.

